# Endogenous Retrovirus 3 – History, Physiology, and Pathology

**DOI:** 10.3389/fmicb.2017.02691

**Published:** 2018-01-15

**Authors:** Yomara Y. Bustamante Rivera, Christine Brütting, Caroline Schmidt, Ines Volkmer, Martin S. Staege

**Affiliations:** ^1^Department of Paediatrics I, Martin Luther University Halle-Wittenberg, Halle, Germany; ^2^Department of Neurology, Martin Luther University Halle-Wittenberg, Halle, Germany

**Keywords:** endogenous viral elements, endogenous retroviruses, ERV3, ZNF117, cancer, autoimmunity, ultra-stability, SOS response

## Abstract

Endogenous viral elements (EVE) seem to be present in all eukaryotic genomes. The composition of EVE varies between different species. The endogenous retrovirus 3 (ERV3) is one of these elements that is present only in humans and other Catarrhini. Conservation of ERV3 in most of the investigated Catarrhini and the expression pattern in normal tissues suggest a putative physiological role of ERV3. On the other hand, ERV3 has been implicated in the pathogenesis of auto-immunity and cancer. In the present review we summarize knowledge about this interesting EVE. We propose the model that expression of ERV3 (and probably other EVE loci) under pathological conditions might be part of a metazoan SOS response.

## Endogenous Viral Elements (EVE)

Several virus species can persist lifelong in their hosts (Norja et al., 2006; Thorley-Lawson et al., 2013). In some cases, persistence is a consequence of integration in the host genome (Wang et al., 2015). In addition to somatic cells, cells of the germ line can be target cells of integration events. The integrated virus can then be transmitted vertically like an ordinary gene (Feschotte and Gilbert, 2012). If such endogenous viral elements (EVE) have no negative effects on the host, EVE can become stable elements of the host genome (Villesen et al., 2004).

Endogenous retroviruses (ERV) are the largest group of EVE and form at least 8% of the human genome (Griffiths, 2001). In some other species the amount of ERV DNA in the genome is much lower, suggesting the existence of efficient control systems in these species (Muir et al., 2004). ERV have been detected in the genomes of virtually all higher eukaryotes (Belshaw et al., 2004; Heidmann et al., 2009). There is growing evidence that ERV have played an important role in the evolution of mammals, primates, and humans (Deininger et al., 2003). Nearly all known human ERV (HERV) integrated up to 100 million years ago (Magiorkinis et al., 2015; Escalera-Zamudio and Greenwood, 2016).

Endogenous viral elements are usually inactivated by genetic and epigenetic mechanisms (Jern and Coffin, 2008). Genetic mechanisms include deletions, inversions, and point mutations in the open reading frames for the viral proteins. Therefore, most EVE are no longer able to replicate and form virus particles autonomously. However, release of virus particles derived from EVE has been described in cancer and other diseases (Wang-Johanning et al., 2007; Volkman and Stetson, 2014). In addition to mutations, epigenetic mechanisms inhibit EVE transcription (Blazkova et al., 2009; Lee et al., 2012). Reactivation of epigenetically silenced EVE can occur and lead to transcription of EVE-encoded proteins or non-coding sequences.

The majority of genomic HERV sequences are incomplete or heavily mutated, are often relatively short, and do not retain the complete retrovirus genome organization. Nevertheless, these HERV-like elements (HERVLE) can contribute to physiological or pathological processes. Complete HERV and HERVLE have been shown to be reactivated in certain types of cancer (Bannert and Kurt, 2004). Reactivated HERVLE can modulate expression of adjacent genes. For instance, HERVLE have been shown to act as alternate promoters for varying cellular genes in Hodgkin lymphoma and Non-Hodgkin lymphoma cells (Huff et al., 2005; Lamprecht et al., 2010; Lock et al., 2014; Babaian et al., 2016).

Endogenous retroviruses have been classified based on sequence similarities, but no system is universally accepted (Blomberg et al., 2009). ERV contain over 200 distinct groups and subgroups. ERV have been classified into three major groups: Class I ERV are related to gammaretroviruses and include human ERVE and ERV3; Class II ERV are related to betaretroviruses and include human ERVK and mouse mammary tumor virus; Class III ERV are related to *Spumaretrovirinae* and include ERVL (Katzourakis and Tristem, 2005).

Endogenous retroviruses are preferentially located on the Y chromosomes of human, chimpanzee and orang-utan (Sin et al., 2010). It has been suggested that reduced recombination of the Y chromosome renders loss of integrated sequences less likely. In addition, the apparently low number of functional genes and the high amount of heterochromatin on the Y chromosome might allow integration of ERV without negative impact (Kjellman et al., 1995).

## The Endogenous Retrovirus 3 (ERV3)

**FIGURE 1:**
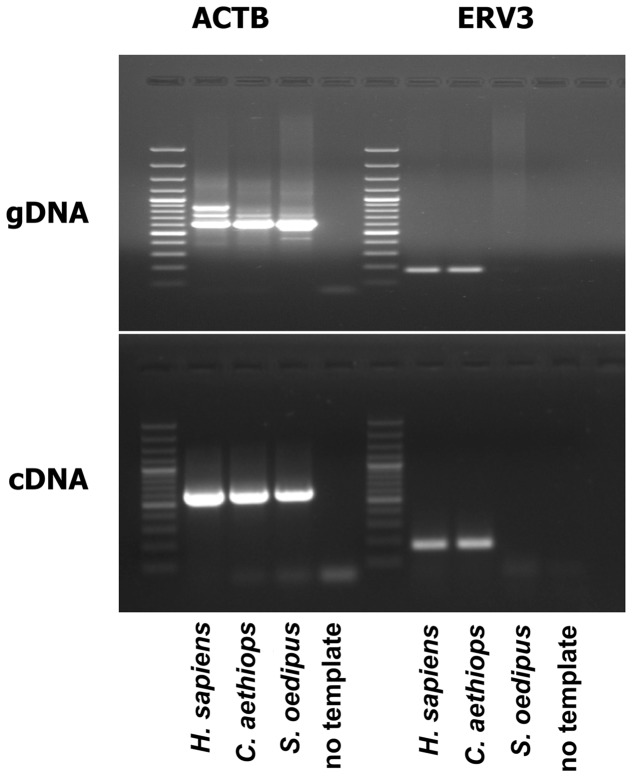
ERV3 is undetectable in cells from *Saguinus oedipus*. Genomic DNA (gDNA) or cDNA from the human Hodgkin lymphoma cell line L-1236 (*Homo sapiens*) (Wolf et al., 1996), the grivet (*Chlorocebus aethiops*) cell line COS-1 (*C. aethiops*) (Gluzman, 1981), and the cotton-top tamarin (*S. oedipus*) cell line B95.8 (*S. oedipus*) (Shope et al., 1973) were used as template for PCR with ERV3 specific primers. Actin beta (ACTB) served as control.

**FIGURE 2:**
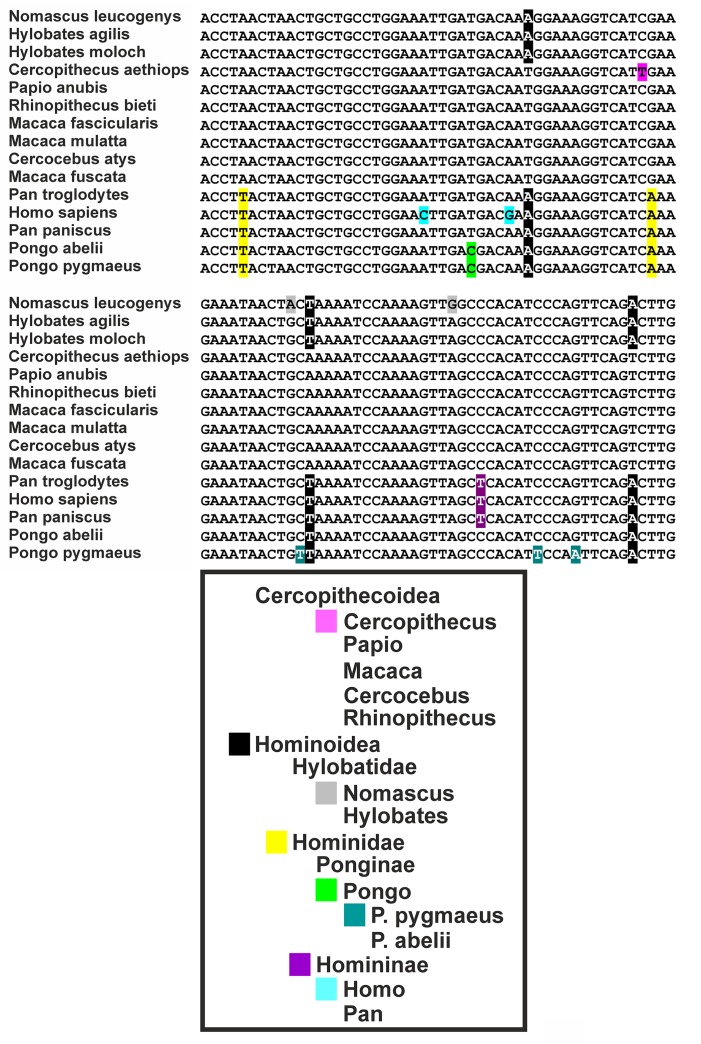
Sequence comparison of ERV3 sequences from different species. Variations specific for individual taxa are highlighted. The following sequences have been analyzed: *Cercocebus atys*: NM_001308247, *Cercopithecus aethiops*: MG574981, *H. sapiens*: NM_001007253, *Hylobates agilis*: AB198937, *Hylobates moloch*: AJ862653, *Macaca fascicularis*: AB198938, *Macaca fuscata*: XM_015446627, *Macaca mulatta*: XM_015133398, *Nomascus leucogenys*: NM_001308194, *Pan paniscus*: XM_014345675, *Pan troglodytes*: XM_016956775, *Papio anubis*: XM_017956681, *Pongo abelii*: NM_001308132, *Pongo pygmaeus*: AB198936, *Rhinopithecus bieti*: XM_017858756.

ERV3 (also known as HERV-R) has been detected only in *Hominidae* (with the exception of *Gorilla*) and *Cercopithecoidea*. ERV3 entered the primate genome obviously 30–40 million years ago, around the time of the separation of the *Catarrhini* and *Platyrrhini* lineages (separation of the Old and New World monkeys). In several studies, ERV3 has been used as marker for the presence of human DNA (Yuan et al., 2001; Whiley et al., 2004; Eberhart et al., 2005; Lee et al., 2005, 2006; Adaui et al., 2006; Rollison et al., 2007; Gage et al., 2011; MacIsaac et al., 2012; Agrawal et al., 2014; Alsaleh et al., 2014; Barletta et al., 2014; Devonshire et al., 2014; Shigeishi et al., 2016). ERV3 is located in great apes, monkeys and humans at an identical genomic position. No ERV3 locus was found in the genome of *Gorilla*. Despite absence of ERV3 from the *Gorilla* genome, sequences with similarity to human ERV3 are present in *Gorilla* (Kim et al., 2006). Indeed, the current Gorilla genome version (gorGor4) contains at least one predicted non-coding gene (LOC109024208) with high sequence similarity to human ERV3. The human genome contains the same non-coding ERV3 copy. In both species, this copy is located upstream of the zinc finger protein ZNF681 on chromosome 19. ERV3 sequences have been found in different species of *Catarrhini* including *Cercopithecinae* (macaques, baboons, mangabyes), *Hylobatidae* (gibbons), and *Hominidae.* No sequences have been found in *Platyrrhini* (Shih et al., 1991; Hervé et al., 2004). As demonstrated in **Figure 1**, ERV3 is detectable at the cDNA as well as genomic DNA level in man (*Homo sapiens, Hominoidea, Catarrhini*; Hodgkin lymphoma cell line L-1236; Wolf et al., 1996) and grivet (*C. aethiops, Cercopithecoidea, Catarrhini;* cell line COS-1; Gluzman, 1981) but not in cotton-top tamarin (*Saguinus oedipus, Cebidae, Platyrrhini;* cell line B95.8; Shope et al., 1973). The ERV3 sequences from *Catarrhini* are highly conserved (**Figure 2**). Unfortunately, a definitive and universally accepted nomenclature for ERV and other EVE has not been established (Mayer et al., 2011; Vargiu et al., 2016). Therefore, several sequences that are annotated in public databases as ERV3 (e.g., gene IDs 71995, 107603642, 105604693, and many others) are not homolog to ERV3 from *Catarrhini.*

**FIGURE 3:**
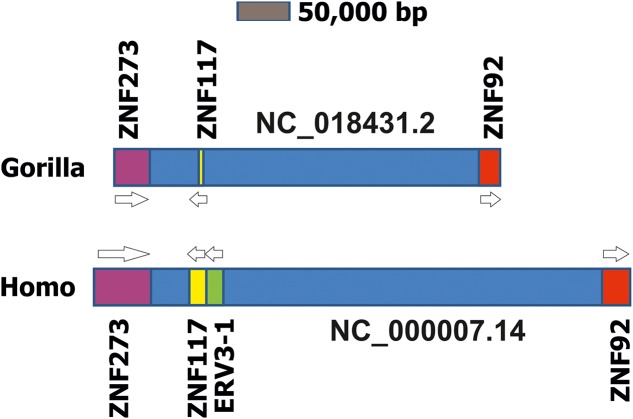
Comparison of the ZNF117 regions in chromosome 7 from *Homo sapiens* and *Gorilla gorilla*. Presented is a schematic drawing of the ZNF117 regions from *H. sapiens* (genome version GRCh38.p7) and *Gorilla gorilla* (genome version gorGor4) on chromosome 7. For both chromosomes the region between the two zinc finger proteins ZNF273 and ZNF92 is presented. Genes are presented as blocks, intergenic regions as dark blue boxes. Homologous gene loci are indicated by identical colors.

ERV3 was isolated from human DNA and cDNA libraries in the mid-80s (O’Connell et al., 1984; Cohen et al., 1985) and named ERV3 because it was the third identified human endogenous retrovirus locus (after ERV1 and virus 51-1). Sequence similarities with mammalian type C retroviruses qualify this ERV as a class I ERV. Human ERV3 is located on chromosome 7 at 7q11 (Kim et al., 2000). Early observations indicated that some of the transcripts from the ERV3 locus contained sequences from the downstream region (Kato et al., 1987). It was found that such transcripts contain sequences from a zinc finger protein (ZNF117) with unknown function (Kato et al., 1990). Interestingly, these read-trough transcripts were more abundant in peripheral blood mononuclear cells (PBMCs) from patients with multiple sclerosis than in PBMC from healthy individuals (Rasmussen et al., 1995). However, a link between the ERV3 locus and multiple sclerosis could not be established (Clausen, 2003). Read-trough transcription from ERV into zink finger proteins seems to be a common theme. For instance, according to nucleotide data bases, ERV-ZNF8 read-trough transcription might occur. Notably, ERV3-ZNF117 read-through transcripts (NM001348050) and normal ZNF117 reference transcripts (NM_015852) encode the identical ZNF117 protein sequence. Therefore, the ERV3 locus can be considered as an alternative promoter for ZNF117. No specific functions for the different untranslated regions of the two transcripts have been identified. According to the RegRNA2.0 (Chang et al., 2013) analysis the shorter 5′-untranslated region of the read-through transcripts might have fewer binding sites for microRNAs and non-coding RNAs. Whether the different ZNF117 transcripts have different stabilities and translation efficiencies should be analyzed. The *Gorilla gorilla* genome contains a sequence with high homology to the human ZNF117 that is located in a predicted gene (LOC101136021, “zinc finger protein 107-like”). In previous genome versions the region was annotated as “zinc finger protein 208-like.” As a consequence of the high number of zinc finger proteins with similar sequences the automated annotation algorithms have obviously not correctly assigned this gene as *Gorilla* ZNF117. However, this homology is evidenced not only by the high sequence similarity but also by the identical chromosomal context (**Figure 3**). Human ZNF117 as well as *Gorilla* ZNF107-like are located on the opposite strand between the two zinc finger proteins ZNF273 (*G. gorilla* LOC101135434) and ZNF92 (*G. gorilla* LOC101137731) on chromosome 7. The sequence between the two zinc finger proteins is remarkably shorter in *Gorilla* than in *Homo* suggesting that the *Gorilla* ERV3 might has been lost by a deletion.

A large proportion of human genomes harbor a polymorphism that results in a truncated ZNF117 protein (Balasubramanian et al., 2011). This single nucleotide polymorphism (rs1404453) introduces a termination codon in the open reading frame resulting in loss of the last 57 amino acids. The putative nucleic acid binding sites are not affected by the truncation. Interestingly, this polymorphism is conserved in other species, suggesting that the shorter protein form might be functionally active.

The human genome contains approximately 40 ERV3-like elements (Kannan et al., 1991; Kjellman et al., 1995; Andersson et al., 2005). Only the copy on chromosome 7q11 has a complete open reading frame for a viral envelope protein; the other open reading frames from this locus are inactivated by non-sense mutations (Kannan et al., 1991). Polymorphisms in the LTR and open reading frame of ERV3 including non-sense mutations that lead to truncated proteins have been observed but no association with diseases has been found (Rasmussen et al., 1996; Rasmussen and Clausen, 1998). Interestingly, approximately 1% of the Caucasian population has mutations in ERV3 that disrupt the open reading frame (de Parseval and Heidmann, 1998). The functional consequences of this inactivation have not been clarified.

ERV3 transcripts are detectable in several normal tissues including lymphoid organs (spleen, lymph nodes, thymus), the gastro-intestinal tract (stomach, duodenum, small bowel, appendix, colon, rectum), the endocrine system (adrenal glands, thyroid), the urinary system (kidney, urinary bladder), placenta, male and female reproductive system (testis, corpus luteum, Fallopian tubes), the respiratory system (lung bronchial epithelium), astrocytes, sebaceous glands, and salivary glands (Larsson et al., 1994; Andersson et al., 1996; Katsumata et al., 1998; Eo et al., 2014; Fei et al., 2014; Kang et al., 2014). The broad expression profile of ERV3 was also found in other species (Schiavetti et al., 2002).

## ERV3 and Immunopathology

The stimulation of the immune system by ERV encoded antigens might be involved in autoimmunity. ERV encoded antigens can be recognized by cytotoxic T cells (Haist et al., 1992). Antibody cross-reactivity between exogenous retroviruses and ERV3 peptides have been described (Katsumata et al., 1999) and ERV3 is up-regulated by cytokines in endothelial cells (Sasaki et al., 2009). Indeed, ERV3 has been suggested as an auto-antigen involved in different immune-pathologies (Takeuchi et al., 1995; Li et al., 1996; de Parseval et al., 1999; Blank et al., 2009; Nelson et al., 2010, 2014; Kowalczyk et al., 2012). Expression of ERV3 was found to be up-regulated in blood cells but down-regulated in skin biopsies from patients with morphea (Li et al., 1996). ERV3 was detected in synovial tissues from patients with rheumatoid arthritis and osteoarthritis but also in synovial tissues of healthy individuals (Nelson et al., 2010). Altogether, the possible involvement of ERV3 in autoimmunity requires further investigations. Like many other retroviral envelope proteins, ERV3 has a functionally active so-called immunosuppressive domain that can reduce immune responses in mice (Mangeney et al., 2007). Immune responses are governed by several host factors including highly polymorphic systems like the major histocompatibility complex. It seems possible that the balance between immunosuppressive and immuno-stimulatory activities depends on the individual combination of such factors.

## ERV3 and Cancer

The role of ERV3 in cancer might vary in different tumor entities. Elevated presence of ERV3 has been detected in colorectal, lung and liver cancer (Ahn and Kim, 2009; Lee et al., 2014). ERV3 is expressed in prostate cancer cells (Wang-Johanning et al., 2003). ERV3 is up-regulated together with other ERV (ERVFRD-1, ERV-PABLB-1, ERVPb-1, ERVV-1, ERVW-1, and ERVW-2) in endometrial carcinoma (Strissel et al., 2012). Besides, ERV3 is co-expressed together with members of the ERVK family and ERVE family in ovarian cancer (Wang-Johanning et al., 2007). Interestingly, 30% of ovarian cancer patients have antibodies against ERV3 whereas such antibodies are not detectable in healthy individuals (Wang-Johanning et al., 2007). This observation underscores the recognition of ERV3 by the immune system. In early studies, ERV3 was not detected in breast cancer (Wang-Johanning et al., 2001). A more recent study observed increased levels of ERV3 in the blood of untreated patients with breast cancer. Levels of ERV3 and other ERV decreased after therapy (Rhyu et al., 2014). Up-regulation of ERV3 in different cancer types might suggest an involvement in the pathogenesis of these diseases.

On the other hand, ERV3 was considered to be a tumor suppressor (Matsuda et al., 1997; Lin et al., 1999, 2000). ERV3 is up-regulated after irradiation of head and neck squamous cell carcinoma cells (Michna et al., 2016), during monocytic differentiation of acute myelogenous leukemia cells (Larsson et al., 1996, 1997; Abrink et al., 1998) as well as during differentiation of normal squamous cells (Otsuka et al., 2006). Demethylation of the ERV3 locus during monocytic differentiation leads to expression of ERV3 and ZNF117 (Andersson et al., 1998). Growth inhibited Hodgkin lymphoma cells express higher levels of ERV3 RNA than proliferating cells (Kewitz and Staege, 2013).

Regulation of ERV3 seems to be cell type specific (Sibata et al., 1997). For instance, ERV3 is up-regulated together with fusogenic ERV envelope proteins in muscle after long-term endurance exercise (Frese et al., 2015). ERV3 is expressed during embryogenesis and a role of ERV3 in developmental processes has been discussed (Andersson et al., 2002). ERV3 expression might be regulated by steroid hormones (Rote et al., 2004). On the other hand, a function of ERV3 in hormone regulation has been suggested (Morrish et al., 2001). In normal placenta, ERV3 is higher expressed in the first trimester of pregnancy than at term (Holder et al., 2012). ERV3 is up-regulated during trophoblast differentiation (Boyd et al., 1993). Like the 5′-long terminal repeats (LTRs) of ERVW-1 and ERVFRD-1, the ERV3 5′-LTR is hypomethylated in cytotrophoblasts during pregnancy (Gimenez et al., 2009). Expression of ERV3 and other ERV in the placenta is reduced in cases of intrauterine growth retardation (Ruebner et al., 2010). The importance of ERV expression in the placenta is, indeed, known for a long time (Muir et al., 2004). An immunosuppressive function of ERV3 in the context of mother-fetus interaction has been proposed (Venables et al., 1995). Other ERV expressed in the placenta have fusogenic activity. Whether ERV3 has fusogenic activity has been discussed controversially (Boyd et al., 1993; Morrish et al., 2001). Together with syncytin 1 and syncytin 2, ERV3 is down-regulated in hydatidiform moles and malignant gestational trophoblastic tumors in comparison to normal placenta (Bolze et al., 2016). ERV3 expression is absent in choriocarcinoma (Cohen et al., 1988; Kato et al., 1988).

Taking together, it seems that in some tumor entities ERV3 is preferentially expressed in differentiated or growth inhibited cells compared to proliferating tumor cells. Whether ERV3 has growth inhibitory activity in certain cell types has to be investigated. Rodent (tumor) models for ERV3 (and other genuine human ERV) have the limitation that ERV3 is not naturally present in these species. Therefore, especially the interaction between immune cells and ERV3 in these models is highly different from the situation that can be expected in the human system. *In vitro* systems might be necessary to reconstruct basic aspects of this interaction. Independent on the function of ERV3 in tumor cells, ERV3 might be considered as target for immunological treatment strategies. The presence of antibodies against ERV3 in some cancer patients indicates that immune responses are possible. Cytotoxic T cells with specificity for ERV3 might be able to kill ERV3 expressing tumor cells. However, the problems of overcoming tolerance on the one hand and avoiding autoimmunity on the other hand have to be solved before ERV3 (which is not a classical cancer antigen) might be useful as immunological cancer target.

## The Metazoan SOS Response

Based on the presented observations, it remains unclear whether ERV3 can act as a tumor suppressor or a tumor promoting factor. It remains possible that the expression of ERV3 in tumor cells has no impact on tumor growth but is only an epiphenomenon related to relaxed gene expression control. ERV3 transgenic rats show no pathology (Tanaka et al., 2003). The limitations of such animal models have been discussed above. The presence of mutations in ERV3 that disrupt the open reading frame in virtually healthy individuals suggest that ERV3 protein has no essential function. In addition, it seems doubtful whether the numerous mutated non-coding copies of ERV3 (and other ERV) have individual functions. We propose a different function for ERV3 and other ERV loci. It was suggested that ERV3 DNA can form a structure that activates the intracellular DNA sensor cyclic GMP–AMP synthase (Herzner et al., 2015). Activation of this enzyme can trigger an inflammatory pathway. The importance of this pathway is highlighted by the development of autoimmunity in patients with defective double-stranded DNA-removal machinery (Stetson et al., 2008). Interestingly, increased ERV expression has been detected in patients with cancer as well as in patients with a spectrum of auto-immune diseases. One of the common features between cancer and auto-immune diseases is the dysfunction of regulatory circuits. Biological systems are characterized by a high level of ultra-stability (Staege, 2014). In cancer cells, normal regulatory circuits are defect. It seems likely that cells have sensor mechanisms that respond to dysfunctional regulatory circuits (DRC). As a consequence of ultra-stability, cells will try to reach alternative steady-state equilibria. The activation of ERV under these conditions might be involved in these mechanisms. DRC can be the consequence of virus infections. If the immune system cannot eliminate this virus directly, the activation of the immune system by EVE can be an alternative pathway that allows elimination of the exogenous virus by varying mechanisms (receptor interference, lysis of EVE-expressing cells by cytotoxic T cells, competition between RNA molecules, and so on and so forth). Such mechanism might be responsible for the detected antibodies against ERV including ERV3 in some cancer patients. This might be one reason why the genomes of virtually all higher organisms contain a plethora of EVE. ERV re-activation in cancer or other diseases can indicate the presence of DRC in these diseases.

In the case of ERV3, loss of ERV3 expression in certain types of cancer can indicate that in these tumors ERV3 expression would otherwise activate the endogenous sensing machinery. The further elucidation of the function of ERV3 and other EVE in health and disease might allow the development of new treatment strategies for cancer and auto-immune diseases.

## Conclusion

ERV3 is a *Catarrhini*-specific EVE with interesting expression profile in normal tissues, cancer and other diseases. ERV3 is closely linked to the neighboring ZNF117 locus and for both genes the physiological function has not been clarified. Differential expression of ERV3 in cancer cells and the corresponding normal tissues makes ERV3 a potential target for future therapeutic developments. However, further investigations are necessary in order to elucidate the role of the ERV3/ZNF117 locus in the context of cancer and other diseases as well as physiological functions of these genes.

## Author Contributions

MS designed the study. IV and CS performed the experiments. All authors analyzed the data, wrote the paper, and approved the final version of the paper.

## Conflict of Interest Statement

The authors declare that the research was conducted in the absence of any commercial or financial relationships that could be construed as a potential conflict of interest.
